# Real-world experience with continuous subcutaneous foslevodopa/foscarbidopa infusion: insights and recommendations

**DOI:** 10.1007/s00702-025-02911-5

**Published:** 2025-03-22

**Authors:** Thomas Koeglsperger, Emir Berberovic, Christian Dresel, Sebastian Haferkamp, Jan Kassubek, Rahel Müller, Christian Oehlwein, Sebastian Paus, Peter Paul Urban

**Affiliations:** 1 Department of Neurology, LMU University Hospital, LMU Munich, Munich, Germany; 2 Department of Translational Brain Research, DZNE-German Center for Neurodegenerative Diseases, 81377 Munich, Germany; 3 Parkinson-Klinik Ortenau, Wolfach, Germany; 4 Department of Neurology, University Medical Center Mainz, Johannes Gutenberg University, Mainz, Germany; 5 Department of Dermatology, University Hospital Regensburg, Regensburg, Germany; 6 Department of Neurology, University Hospital Ulm, Ulm, Germany; 7 Department of Neurology, University Hospital Augsburg, Augsburg, Germany; 8 Neurology Private Practice, Gera, Germany; 9 Department of Neurology, GFO Kliniken Troisdorf, Troisdorf, Germany; 10 Department of Neurology, Asklepios Klinik Barmbek, Hamburg, Germany

**Keywords:** Parkinson’s disease, Motor Fluctuations, Advanced Treatment, Foslevodopa/Foscarbidopa

## Abstract

Traditional advanced therapies in Parkinson’s disease (PD) with motor fluctuations and dyskinesias like continuous apomorphine infusion (CSAI), levodopa-carbidopa intestinal gel (LCIG), levodopa-carbidopa entacapone intestinal gel (LECIG), or deep brain stimulation (DBS) have played a central role in managing therapy-related complications. Recently, continuous subcutaneous foslevodopa/foscarbidopa infusion (CSFLI) has emerged as a novel therapeutic option. This manuscript provides insights from one year of real-world experience with CSFLI, addressing critical questions that clinicians face when selecting the most appropriate therapy for advanced PD. Our discussion centers on key considerations for patient selection, exploring which individuals may benefit more from CSFLI compared to other device-aided therapies. We highlight CSFLI’s advantages in flexibility and ease of use but also consider limitations, particularly its side effects, such as skin-related issues. Recommendations are presented on how to prevent and manage these adverse effects to maximize patient compliance and therapeutic success. Additionally, the paper examines strategies for optimizing concurrent oral medications when combined with CSFLI, providing guidance on balancing pump infusion with necessary adjunctive oral treatments.

## Introduction

### Definition of motor and non-motor fluctuations in PD

PD is characterized by progressive symptoms, including both motor (MS) and non-motor symptoms (NMS), which vary throughout the day or from day to day (Poewe et al. 2017). Levodopa therapy, the primary treatment for PD, is linked to fluctuations such as motor fluctuations (MF) and non-motor fluctuations (NMF), causing a narrowing of the therapeutic window. These fluctuations occur when medication effectiveness diminishes, resulting in periods of both hypokinesia (resurgence of MS and NMS) or dyskinesia. MF affect 80% of patients after 10 years, typically beginning around three years post-diagnosis, and severely impact patients' quality of life (QoL) (Denny and Behari 1999). Common medication-related fluctuations include wearing OFF, where MSs and NMSs return before the next dose, often requiring more than five doses daily, thus complicating daily activities (Leta et al. 2021). Dyskinesias, involuntary movements caused by levodopa, include peak-dose dyskinesias, OFF dyskinesias, and biphasic dyskinesias, with peak-dose dyskinesias being the most common. The prevalence increases with levodopa use, affecting up to 54% of patients after 3–6 years, though disabling dyskinesias are less frequent (Investigators PSGCC 2009). These movements reduce QoL and increase healthcare costs (Hung et al. 2010; Winter et al. 2011; Hechtner et al. 2014). NMS such as sleep disturbances, pain, and gastrointestinal symptoms were first described by James Parkinson in 1817 (Parkinson 2002; Fahn 2015). These symptoms affect 60%–97% of PD patients and often fluctuate similarly to MS, a phenomenon known as non-motor fluctuations (NMF) (Martinez-Martin et al. 2007). NMF are typically categorized into neuropsychiatric, dysautonomic, and sensory fluctuations (Hillen and Sage 1996).

### Pharmacological treatment of MF

MF in PD are managed with various medication-based and interventional therapies, guided by frameworks like MANAGE PD and CEDEPA (Luquin et al. 2017; Ebersbach and Poewe 2018; Fabbri et al. 2023; Fernandez et al. 2023). Initial treatment often involves adjusting levodopa dosing intervals or adding soluble formulations for quicker onset, while additional options like monoamine oxidase-B (MAO-B) or catechol-O-methyl transferase (COMT) inhibitors may reduce OFF times if control remains insufficient (Ferreira et al. 2016, 2019; Lees et al. 2017). Novel on-demand treatments such as subcutaneous or sublingual apomorphine and inhalable levodopa offer flexible responses to sudden OFF episodes (Pahwa et al. 2023). Invasive treatments for PD, like DBS and pump-based therapies, should be considered for patients requiring frequent levodopa doses (≥ 5 times daily), experiencing prolonged OFF symptoms, or disruptive dyskinesias. Decisions should involve QoL scores (e.g., PDQ-39) and follow insufficient response to combined medication approaches (Höglinger 2023).

## Summary of the evidence for CSFLI in the treatment of PD.

CSFLI employs a novel formulation of levodopa and carbidopa prodrugs, delivered via a continuous subcutaneous infusion for the treatment of advanced PD with motor fluctuations. A Phase 1 open-label, randomized, 2-period crossover study comparing the pharmacokinetics of CSFLI to LCIG demonstrated that the levodopa exposures following a 24-h subcutaneous infusion of CSFLI were like those of LCIG over 16 h, supporting the potential of CSFLI to provide consistent levodopa exposure throughout the day (Rosebraugh et al. 2021, 2022). The safety and efficacy of CSFLI was assessed in two key clinical trials. The first, a 12-week Phase 3, randomized, double-blind trial, compared CSFLI with oral levodopa (Soileau et al. 2022). The results showed that CSFLI significantly increased ON time without troublesome dyskinesia (mean difference 1.75 h) and reduced OFF time (mean difference −1.79 h) compared to oral levodopa. The CSFLI group also had higher rates of infusion site adverse events (erythema, pain, cellulitis), but these were mostly non-serious and mild to moderate in severity. Despite a higher discontinuation rate (22% vs. 1% for oral levodopa), the overall benefit-risk profile favored CSFLI. A second, 52-week open-label trial further evaluated the long-term safety and efficacy of CSFLI in patients with advanced PD (Aldred et al. 2023). Over 52 weeks, patients receiving CSFLI showed significant improvements in normalized ON time without troublesome dyskinesia (mean change 3.8 h) and OFF time (mean change −3.5 h). Additionally, the percentage of patients experiencing morning akinesia decreased from 77.7% at baseline to 27.8% at week 52. Improvements in sleep quality and QoL were also observed. Although infusion site reactions remained the most common adverse event, the treatment was generally well tolerated. In conclusion, CSFLI offers a promising non-surgical treatment for advanced PD, improving motor fluctuations and QoL with a favorable benefit-risk profile.

## CSFLI in clinical practice: key challenges and consideration

### Comments on the indication

Levodopa uptake may be hindered in PD due to delayed pharyngeal, gastric and intestinal motility (Merims et al. 2003; Pfeiffer 2018; Labeit et al. 2022), competition with the absorption of proteins and other medications (e.g., iron supplements, antacids), as well as malabsorption. When comparing oral (p.o.) and subcutaneous (s.c.) therapies, s.c. administration may thus offer advantages. S.c. delivery bypasses the gastrointestinal tract, enabling more consistent drug absorption and stable plasma levels, which may improve patient response rates to levodopa (Rosebraugh et al. 2021, 2022). This prompts consideration of whether the current clinical standard levodopa test should be replaced with a subcutaneous levodopa test using CSFLI. The levodopa test evaluates improvement in the Unified Parkinson's Disease Rating Scale (UPDRS) motor Part III. A > 30% improvement suggests PD, while < 30% indicates non-PD (Höglinger 2023). Levodopa-naive patients typically receive 10 mg domperidone thrice daily before testing, followed by 150–250 mg levodopa after a > 12 h. interval. Subcutaneous apomorphine is an alternative but causes more side effects and requires titration (Merello et al. 2002; Höglinger 2023). Studies show levodopa test sensitivities of 70–77% and specificities of 63.9–81.4%, with improvements ≥ 16% strongly suggesting PD (Rossi et al. 2000; Merello et al. 2002). Combining levodopa testing with hyposmia detection raises sensitivity to 90% and specificity to 74%. However, chronic levodopa therapy provides higher diagnostic accuracy but delays results (Chambi et al. 2017). Levodopa response also guides advanced therapies. For instance, according to German PD guidelines, DBS is recommended for PD patients with ≥ 33% improvement in MS via levodopa test, along with other criteria (Höglinger 2023). Nonetheless, gastrointestinal absorption issues may affect levodopa test sensitivity (Nutt et al. 1984; Leenders et al. 1986). Thus, any measure to increase the tests sensitivity would have extensive clinical implications. We thus believe that future studies should address the validity of a s.c. levodopa test and compare its sensitivity and specificity to the standard p.o. test.

Another key question concerns specific patient subgroups that could benefit most from s.c. therapy. In an earlier stage of the disease, some patients might prefer CSFLI over p.o. medication due to its convenience and the increased independence it offers from meal-related considerations. However, given the significantly higher incidence of side effects, including skin reactions (Soileau et al. 2022), as well as the substantial cost of CSFLI, we strongly advise against its use solely for reasons of convenience. In a subgroup of patients suffering from nocturnal akinesia, a purely nocturnal application could be an effective approach. Furthermore, CSFLI may have a future role in the treatment of NMS and their fluctuations. Among NMS, neuropsychiatric symptoms—particularly depression and anxiety—are the most frequent and impactful. They often reduce QoL more than motor fluctuations (Witjas et al. 2002; Storch et al. 2013; Stocchi et al. 2014; Rieu et al. 2016; Franke and Storch 2017). Some neuropsychiatric fluctuations are linked to dopaminergic states and many autonomic symptoms, such as urinary urgency, and sensory symptoms, including pain, might be associated with OFF periods (Nebe and Ebersbach 2009; Storch et al. 2013). Although other symptoms, such as breathlessness and cardiovascular symptoms, do not consistently align with MF and are minimally impacted by chronic levodopa therapy, maintaining consistent levodopa plasma concentrations would likely help mitigate some NMFs. We therefore recommend that future studies investigate the potential of CSFLI to alleviate NMFs and its overall impact on QoL in PD. In selected patients with nocturnal akinesia the question arises whether a purely nocturnal application could be an effective approach.

Continuous treatment in patients with impulse control disorder (ICD), or levodopa-associated side effects such as hallucinations or psychosis, is controversial. Previous studies (Soileau et al. 2022) demonstrated a higher rate of hallucinations in CSFLI compared to p.o. treatment, suggesting that s.c. treatment comes with an increased risk for psychiatric side effects. Conversely, transitioning from pulsatile p.o. intake to continuous levodopa delivery may offer significant benefits. For example, non-compliant patients who exceed the recommended levodopa dosage might achieve improved symptom control with CSFLI compared to p.o. therapy, potentially reducing side effects. Future studies should focus on identifying specific clinical characteristics and patient subgroups that predict which approach—s.c. or p.o.—would be most suitable for individual patients.

### CSFLI: expanding the spectrum of continuous drug delivery for PD

The development of CSFLI adds to the growing portfolio of different applications of levodopa to PD which initially comprised only oral administration, including immediate release and controlled release together with dispersible and soluble levodopa formulations. The classical oral levodopa is still in further optimization, e.g. by the addition of IPX203 as an oral extended-release levodopa/carbidopa formulation that was designed to prolong levodopa plasma concentrations (Espay et al. 2024a). As a non-invasive, levodopa-based, on-demand application to treat OFF episodes in PD, levodopa inhalation powder has been introduced, administered with a breath-actuated inhaler device for pulmonary delivery to provide a fast treatment effect, independent of gastrointestinal dysfunctions or food intake (Jost et al. 2023b). Prior to CSFLI, non-oral/device-aided continuous levodopa delivery therapy, approved for PD patients, comprised LCIG/LECIG for several decades, i.e., fine particles of levodopa/carbidopa dispersed in a methylcellulose gel and delivered using a pump through a percutaneous gastrostomy (PEG) tube with an extension tube so that the gel is released within the jejunum as the primary site of levodopa uptake (Dean and Standaert 2024). As a variant device-aided levodopa-based therapy option in advanced PD, the triple combination levodopa–entacapone–carbidopa intestinal gel (LECIG) has been developed to increase the bioavailability of levodopa from the infusion and has demonstrated to be effective and safe when managed under real clinical practice in a retrospective observational study (Santos-García et al. 2025). Continued research in LCIG has found additional utility and benefits in the treatment of both motor and NMS in PD (Dean and Standaert 2024) and with respect to the effects of long-term LCIG treatment, the DUOGLOBE study (Chaudhuri et al. 2023) demonstrated benefits to OFF time and other parameters to persist for more than 3 years and the COSMOS study showed continued efficacy for 5 years and more (Fasano et al. 2023). Such long-term data cannot exist yet for CSFLI due to its recent introduction, but the differential indication for these therapies will now be an element of advanced PD therapy. It is apparently intuitive that minimally invasive, subcutaneous administration is less burdensome and less challenging than the surgical implantation and the constant care for a PEG tube, especially but not limited to the post PEG placement period (Antonini et al. 2017). However, the PEG may be needed for food and fluid supply in multimorbid, aged PD patients as well. In addition, given that the most frequently reported adverse events for CSFLI are related to infusion site reactions (Soileau et al. 2022), local skin- and lack of subcutaneous bodyfat-related factors in cachectic advanced PD patients might limit the applicability of CSFLI so that nursing considerations will play a role. Moreover, the differential therapy indication in advanced PD will further expand to different subcutaneous levodopa infusion options. In 2024, ND0612 (a solution of levodopa and carbidopa) met the primary endpoint of significantly improving On time without troublesome dyskinesias (1.72 h) in the randomized, double-blind, double-dummy, active-controlled phase 3 trial BouNDless (Espay et al. 2024c) and also significantly reduced OFF time (−1.4 h) and led to significant improvement in the MDS-UPDRS Part II and Patient and Clinical Global Impression of Change rating scales. In summary, both CSFLI and ND0612 were efficacious in treating MS in PD patients in their phase 3 clinical trials. In addition, DIZ102 has been introduced, i.e., levodopa plus carbidopa consisting of a stock solution where both are brought to a physiologically favorable pH of 5.0–5.3 by continuous mixing with a buffer, together with DIZ101, a levodopa/carbidopa solution for intravenous administration which differs from DIZ102 only in the composition of the buffer (Bergquist et al. 2022). Both solutions were shown to have an equally good motor efficacy as LCIG in a clinical pilot study in 18 PD patients (Bergquist et al. 2024). All these levodopa-based device-aided therapies will be compared to the longstanding dopamine agonist-based therapy option of continuous subcutaneous apomorphine infusion (CSAI) (Kukkle et al. 2023). With this current (and growing) portfolio of continuous device-aided levodopa therapies, the therapeutic decisions in advanced PD will probably include more individualized approaches tailored to each given patient´s specific needs (Schröter et al. 2024).

### Managing side effects

#### Skin lesions and their treatment

Patients treated with CSFLI as a new soluble formulation of levodopa and carbidopa prodrugs delivered by a 24 h/day continuous subcutaneous infusion may present with different forms of skin irritation. In the phase III randomized controlled trial by Soileau et al., the most frequently reported adverse events in the CSFLI group were infusion site reactions, affecting 72% of patients compared to 12% in the control group (Soileau et al. 2022). These reactions encompassed a wide range of symptoms, including erythema (27%), pain (26%), cellulitis (19%), edema (12%), bruising (8%), hemorrhage (8%), nodule formation (8%), infection (5%), pruritus (5%), and swelling (5%). Most adverse events were mild or moderate in severity and not considered serious. However, two participants reported serious infusion site infection adverse events (infusion site cellulitis and catheter site cellulitis) in the CSFLI group that required treatment with antibiotics. None of the infusion site adverse events resulted in systemic complications. Importantly, similar cutaneous side effects have been observed with varying frequency and severity during continuous subcutaneous administration of apomorphine indicating that both agents can trigger an inflammatory response (Carbone et al. 2019). To gain a deeper understanding of the pathophysiology, punch biopsies from infusion site reactions in patients treated with CSFLI have been analyzed. Histopathologic examinations revealed lymphocyte-dominant and neutrophil-rich inflammatory infiltrates in the deep dermis and adipose tissue (Yoshihara et al. 2024; Weise and Haferkamp 2025) whereas cutaneous reactions to subcutaneous administration of apomorphine are associated with an eosinophil-rich inflammatory infiltrate (Acland et al. 1998). The observations related to infusion site adverse events from the RCT have been confirmed in the first months of practical experience in the real-world setting. Moreover, algorithms for the management and prevention of cutaneous side effects have been developed to improve QoL and limit disruption of subcutaneous therapy. First, meticulous hygiene at the site of skin perforation by the needle is essential to prevent complications. Regular skin care, such as the application of moisturizing creams twice daily, is recommended. Furthermore, we might advise our patients to change the needle daily or every other day if they are comfortable with this procedure. In some patients, daily needle change was necessary to avoid the occurrence of infusion site nodules. For skin irritations, topical treatment with potent corticosteroid, such as mometasone furoate (0.1%), may be beneficial and is recommended due to its efficacy and favorable safety profile (Spada et al. 2018; Mendes-Bastos and Simoes 2024). Antibiotic treatment should be considered if there are signs of systemic inflammation (fever, increased leukocyte count, or elevated CRP) or if the skin irritation does not respond to topical steroids.

#### OFF phases

In the Phase III RCT (Soileau et al. 2022), a smaller proportion of participants in the CSFLI group reported being Off upon waking compared to the p.o. levodopa–carbidopa group (17% vs. 63%), even though the latter group had the option for nighttime dosing if needed. In our experience, OFF periods can be effectively managed by increasing the base infusion rate. Persistent MF may be further improved by combining treatment with opicapone, which also enables a reduction of the infusion rate of CSFLI. Interestingly, some CSFLI patients reported postprandial OFF episodes, which were managed using an extra bolus. Postprandial OFF periods, despite stable plasma concentrations, may be in part caused by competition between levodopa and amino acids at the blood–brain barrier (Leenders et al. 1986). However, the underlying pathophysiology of postprandial OFF in the context of parenteral administration remains unclear.

#### Dyskinesia

The ON time without dyskinesia increased by 25% from baseline in the CSFLI group, compared to a 7% increase in the p.o. levodopa–carbidopa group, as a percentage of the waking day (Soileau et al. 2022). Dyskinesias can be managed by adjusting the base rate and, when necessary, combining with amantadine.

#### Falls

Falls were reported in 8% of participants in the CSFLI group and 18% in the oral levodopa–carbidopa group (Soileau et al. 2022). To date, we have observed falls only in the context of orthostatic dysregulation and freezing, which, in our opinion, are not causally linked to the CSFLI treatment.

#### Psychiatric side effects

Hallucinations or psychosis events were reported in 15% of participants in the CSFLI group and 3% in the levodopa–carbidopa group. In most participants in each treatment group (2 of 2 in the oral levodopa–carbidopa group and 9 of 11 in the CSFLI group), these events were non-serious and mild or moderate in severity, with no action taken regarding the study drug (Soileau et al. 2022). Consistent with findings in the Phase III trial, hallucinations and psychosis have not been identified as specific phenomena associated with CSFLI treatment.

### Combining oral medication with pump therapy

CSFLI has been approved for the advanced stage of PD when oral therapies fail to sufficiently control motor fluctuations. Due to its continuous application, CSFLI monotherapy for PD symptoms is possible and, in most cases, sufficient. In addition, CSFLI can be combined with (existing) oral PD medication. In that case, it is recommended to check and adjust the dosing of the dopaminergic co-medication to avoid overdosing and dyskinesias. One major argument to use continuous infusion of levodopa instead of oral application is the unreliable uptake of orally administered levodopa in the gastrointestinal tract in advanced PD that leads to variable levels of levodopa in the serum and hence motor fluctuations as well as OFF periods (Merims et al. 2003; Pfeiffer 2018; Labeit et al. 2022). These pharmacokinetic characteristics need to be considered when combining CSFLI with oral dopaminergic therapies. CSFLI has been approved for PD therapy earlier in Europe than in the US, and no specific recommendations have been provided regarding mono- or combination therapy.

The initial proof-of-efficacy studies tested singular CSFLI therapy against oral levodopa with no oral recue treatments allowed (Rosebraugh et al. 2021, 2022). Meanwhile, a follow-up single arm, open label phase 3 study on 137 PD patients allowed the concomitant intake of oral dopaminergic medication (Aldred et al. 2023). The authors did not report any complications when combining CSFLI to the patients’ previous dopaminergic medication. The study drug (i.e., CSFLI) was discontinued in 11% of patients due to hallucinations (4%) or dyskinesias (2%). However, it is unclear if these (most likely) levodopa-related side effects were induced by CSFLI, the oral intake of other dopaminergic therapies or the combination thereof. In a large randomized, controlled, phase III trial on continuous subcutaneous levodopa infusion, ancillary oral levodopa intake was allowed and did not impose additional risks or undesired medication effects to the PD patients (Espay et al. 2024c). To date, no specific recommendations on the use of oral dopaminergic medication in addition to CSFLI therapy or CSFLI combination therapies have been published. In the personal experience of the authors, the therapeutic approach depends very much on the individual patient, his/her clinical stage, previous undesired effects of oral medication, his/her personal preferences and his/her social support. Unpredictable OFF periods, dyskinesias, gastrointestinal problems, adherence issues and a patient’s aversion against taking too many pills per day argue for a continuous dopaminergic stimulation by CSFLI. By contrast, frequent skin irritations, difficult titration of the flow rate of the CSFLI pump, frequent changes of motor requirements (e.g., for sport activities) and previous benefit of other medications such as amantadine propose a combined application of oral and infusion therapies.

Small case series and retrospective studies reported an additional benefit for PD patients who received device aided continuous levodopa delivery as a rescue therapy after deep brain stimulation (Boura et al. 2021; Pürner et al. 2023). The treatment with LCIG/LECIG significantly reduced motor fluctuations and OFF time and increased the quality of life in DBS-treated PD patients. However, to date, there is no indication for a combination of DBS and LCIG/CSFLI in the first place. Although not designed (and not approved) for that purpose, CSFLI can serve as a temporary dopaminergic treatment in specific circumstances, e.g., for managing an akinetic crisis (see Sect. 3.6.), or bypassing the period before an invasive procedure (e.g., implantation of DBS electrodes). Case reports demonstrated the safety and efficacy of such an approach (Loeffler et al. 2024). Although, no systematic study has been done yet, we and other authors (Pötter-Nerger et al. 2024) believe that CSFLI is a viable option in these circumstances, particularly in countries, where no intravenous formulation of levodopa is available. In summary, a systematic study on the actual use, safety and benefits of combining CSFLI with other PD therapies would be a valuable guidance for physicians treating advanced stage PD.

### CSFLI dosing: converting oral medication dosing to a pump infusion rate

#### Dosing scheme

When initiating CSFLI therapy, levodopa equivalent (LE) is calculated based on the patient’s previous daily oral levodopa dose, including adjustments for COMT inhibitors. According to AbbVie’s recommendations, the LE is calculated by multiplying the total dose of immediate-release levodopa, including enteral suspension levodopa, by 1. For sustained-release, controlled-release, or prolonged-release levodopa, the dose is multiplied by 0.75. If COMT inhibitors are being used, the sum of the calculated LE is further multiplied by 1.33. The calculated LE can be used to determine the infusion rate by either consulting the dosing table provided by the drug manufacturer or applying the formula: ${\text{Hourly infusion rate }}\left( {\frac{{{\mathrm{ml}}}}{{\mathrm{h}}}} \right) = { }\left[ {\frac{{\left( {{\text{LE }} \times { }0.92{ } \times { }1.41} \right)}}{240}} \right] \div {\text{ X}}$, where X represents the patient’s total awake time (hrs.) (AbbVie, Dosing and Administration of Produodopa™). Of note, dopamine agonists, MAO-B inhibitors, and glutamatergic drugs are excluded from AbbVie’s conversion, which may contribute to discrepancies between the calculated dose and the patient’s actual requirement. In such cases, the levodopa equivalent daily dose (LEDD) can be determined using established conversion methods (Jost et al. 2023a). Although the calculated infusion rate provides a useful starting point, adjustments are often necessary during the first few days of therapy. In our experience, the initial dose frequently falls at the lower end—or even below—the clinically effective range, necessitating an increase in the hourly infusion rate. This discrepancy may stem from pharmacokinetic differences with CSFLI, which lacks the peak plasma levels achieved with oral medication. Other contributing factors include the exclusion of additional antiparkinsonian drugs in AbbVie’s dosing scheme (unless continued orally), previously low levodopa doses limited by dyskinesia risk, interactions with residual dopaminergic medications, or variability in the skin's absorption of CSFLI. Individual differences in skin histology and characteristics may further influence absorption.

#### Causes for non-response

Despite careful monitoring and adjustments to the infusion rate, some patients do not achieve satisfactory improvement. In our experience, the most important explanation is an incorrect diagnosis: patients may be misdiagnosed with idiopathic PD with MF based on their history yet suffer from atypical PD and are not fully responsive to levodopa. We strongly recommend extended clinical observation or proper home monitoring using suitable motion sensors (Pfister et al. 2020; Fietzek et al. 2023) to accurately determine whether MFs are present.

Persistent symptoms despite appropriate dosing may include PD symptoms that are non-responsive to levodopa, NMS, or tremor. For instance, postural (axial) symptoms, such as camptocormia (Ou et al. 2018; Huh et al. 2022) often respond poorly—or not at all—to levodopa in advanced stages of the disease (Margraf et al. 2016; Imbalzano et al. 2023). Currently, no specific pharmacological treatment exists for postural abnormalities. However, several small studies have shown improvement in subacute axial postural disorders following the discontinuation of dopamine agonists, suggesting a possible relationship (Artusi et al. 2023). In such cases, tapering dopamine agonists while compensating with an equivalent dose of CSFLI should be considered.

Although CSFLI helps maintain constant plasma concentrations (Rosebraugh et al. 2021, 2022), MFs, including dyskinesia, may persist in some patients. This persistence can be attributed to the fact that not all MFs are directly associated with levodopa, and motor states can fluctuate independently of medication intake due to various reasons (Ebersbach et al. 2024).

For these patients, adjunct treatment with amantadine may be beneficial due to its proven efficacy against dyskinesia (Caroff et al. 2020; Espay et al. 2024b). Alternatively, safinamide at 100 mg/day has shown promise as a combination therapy option for dyskinesia management (Cattaneo et al. 2015). PD tremor often shows a less consistent response to levodopa therapy compared to rigidity or bradykinesia (Pirker et al. 2023). For persistent tremor, additional oral therapies—such as beta blocker, anticholinergic drugs, clozapine, or dopamine agonists— may be considered based on the patient’s individual risk profile. Alternatively, deep brain stimulation or MRI-guided focused ultrasound can be effective, powerful treatment options for refractory tremor (Lin et al. 2021).

During the reduction of dopaminergic medications, an increase in NMS is sometimes observed. Care should be taken to taper dopamine agonists slowly, as rapid reduction can exacerbate NMS and potentially lead to dopamine agonist withdrawal syndrome (Rabinak and Nirenberg 2010). In such cases, reinstating the last effective dose of the dopamine agonist is recommended, followed by a very gradual taper if necessary.

Postprandial OFF episodes can occasionally occur during CSFLI therapy. It is essential to rule out postprandial hypotension, often associated with orthostatic hypotension in PD patients, as it can worsen PD symptoms (Chaudhuri et al. 1997). In such cases, therapeutic strategies like meal fractionation, adequate fluid intake, a high-salt diet, or pharmacological treatments such as midodrine or droxidopa should be considered (Seppi et al. 2019).

### The role of CSFLI in emergencies (e.g., akinetic crisis)

The akinetic crisis is an acute, potentially life-threatening worsening of symptoms in PD patients. It manifests as a transient, symptom complex characterized by severe akinesia, rigidity, dysphagia, autonomic symptoms, and fever (Takubo et al. 2003; Onofrj and Thomas 2005). Key diagnostic criteria include acute symptom worsening, such as a > 20-point increase on the UPDRS, and transient resistance to dopaminergic medications for over three days (Thomas and Onofrj 2005). The condition is often linked to infections, medication changes, trauma, or other stressors (Wang et al. 2022). Symptoms include fever, altered consciousness, confusion, appetite loss, dysphagia, autonomic dysfunction, and occasionally myoclonus (Takubo et al. 2003; Onofrj and Thomas 2005). Laboratory findings often reveal elevated creatine kinase, myoglobin, and inflammatory markers (Kuno et al. 1997; Takubo et al. 2003). Risk factors include advanced PD stages, hallucinations, dementia, and motor fluctuations.

The management of akinetic crisis is challenging due to severe dysphagia, gastrointestinal motility issues, and transient Levodopa resistance, limiting oral therapy and increasing complications (Wang et al. 2022). Early and adjusted interventions are crucial for improving outcomes. Current recommendations, based on 23 observational studies due to the lack of randomized trials, emphasize the importance of addressing triggers like infections (antibiotics for pneumonia or UTIs), correcting electrolyte imbalances, and stopping triggering drugs such as neuroleptics. Supportive care includes regular monitoring of vital signs, antipyretic measures, thrombosis prophylaxis, and intravenous hydration (Wang et al. 2022). Dopaminergic therapies play a central role. Levodopa, delivered via nasogastric or PEG tubes, requires dose adjustments for transient resistance (Gordon and Frucht 2001). Alternatives include apomorphine, effective in some cases via subcutaneous infusion, though limited by transient non-responsiveness and mortality rates of 15–21.4% (Bonuccelli et al. 1992; Kipps et al. 2005). Transdermal Rotigotin, starting at 2 mg/24 h and titrated to 6 mg/24 h, shows symptom improvement in severe dysphagia cases (Dafotakis et al. 2009; Fiore et al. 2014). Non-dopaminergic treatments like intravenous amantadine target antiglutamatergic pathways during Levodopa-resistant phases (Kornhuber et al. 1993; Greulich and Fenger 1995).

CSFLI may be a suitable add-on therapy or alternative to the medications indicated above. In theory, it provides sufficiently high and stable levodopa plasma concentrations (Rosebraugh et al. 2022). In a case of a 78-year-old patient with acute akinesia due to dopamine-sensitive hydrocephalus, off-label CSFLI was successfully administered after other options failed (Loeffler et al. 2024). Continuous foslevodopa/foscarbidopa improved motor scores significantly, demonstrating its safety and effectiveness as a potential treatment for akinetic crises. However, the diagnostic context (“dopamine-sensitive hydrocephalus”) requires further investigation in a well-defined patient population. Notably, in an intensive care setting, and in cases where the pump fails (e.g., due to battery issues), CSFLI can be administered via a readily available perfusor connected to subcutaneous access, offering a practical alternative to commercially available pump devices.

### Differential indication

While all device-assisted therapies (DATs) incl. CSFLI are approved for the treatment of motor fluctuations, patient profiles vary significantly, necessitating an individualized approach for differential indication (Table 1). Therapy selection should be personalized based on effectiveness, tolerability, and patient preference, considering factors like disease duration, symptoms, cognitive function, and caregiver support (Reese et al. 2025). For instance, while mild cognitive impairment (MCI) does not exclude a patient from receiving DBS, dementia is generally considered a contraindication although no definitive cutoff or specific test has been established to determine whether DBS should be preferred over pump therapies. (Abboud et al. 2015; Barbosa and Fichman 2019; Bove et al. 2020; Xie et al. 2022). Likewise, severe depression, suicidality, and psychosis contraindicate DBS (Moro and Lang 2006). Conversely, if dopamine overmedication leads to psychiatric side effects, STN-DBS is the only therapy that enables medication reduction, though frontal dysfunction may predict poor long-term outcomes in such cases (Cavallieri et al. 2021). DBS can also benefit patients with impulse control disorders (Lhommée et al. 2012, 2018; Healy et al. 2022), but LCIG has also shown promise for these patients (Catalan et al. 2018; Lopiano et al. 2019). Currently, no data exist on the effects of CSFLI in impulse control disorders.

**Table 1:** Comparison of Advanced Therapies for Parkinson’s Disease: Efficacy, Safety, and Patient Considerations

Feature	CSFLI (Continuous Subcutaneous Foslevodopa/ Foscarbidopa Infusion)	LCIG/LECIG (Levodopa-Carbidopa ± Entacapone Intestinal Gel)	CSAI (Continuous Subcutaneous Apomorphine Infusion)	DBS (Deep Brain Stimulation)
Mode of delivery	Continuous subcutaneous infusion via tube, pump	Continuous intrajejunal infusion via PEG-J tube, pump	Continuous subcutaneous infusion via tube, pump	Implanted brain electrodes delivering electrical stimulation, generator
Efficacy in motor fluctuations	Significant reduction of OFF-time and dyskinesia	Significant reduction of OFF-time and dyskinesia	Significant reduction of OFF-time and dyskinesia	Significant reduction of OFF-time and dyskinesia
Efficacy in non-motor symptoms	Potential benefits, but more data needed	Improves sleep, mood, and gastrointestinal function	Improves sleep, mood, perception, attention, memory, excessive sweating and urogenital function	Improves sleep, mood, cognition, perception, urogenital function, and excessive sweating
Side effects	Frequent (> 50%) Infusion site reactions (nodules, erythema), dyskinesia, nausea	GI complications (e.g., tube displacement, infection), dyskinesia	Skin nodules, hypotension, nausea, psychiatric effects (hallucinations, impulse control disorders)	Surgical risks, potential cognitive effects, speech and gait impairments difficulties
Invasiveness	Minimally invasive (subcutaneous infusion)	Requires PEG-J tube placement	Minimally invasive (subcutaneous infusion)	Requires brain surgery
Patient selection	Advanced PD patients with motor fluctuations not well controlled with oral therapy	Advanced PD patients with motor fluctuations; when patients require a GI tube for other reasons	Advanced PD Patients with motor fluctuations not well controlled with oral therapy and who tolerate apomorphine	Best for younger patients with disabling motor fluctuations and no severe cognitive impairment
Long-term safety data	No long-term data available	Established long-term safety profile	Long-term use feasible but limited data compared to LCIG	Best long-term data profile available
Role in emergency situations (e.g., akinetic crisis)	Potential role, but more data needed	Possible use but requires PEG-J	Can be used acutely in rescue situations	Not an acute treatment option

DBS, CSAI, and LCIG are likely similarly effective for the treatment of motor symptoms, except tremor, which typically responds better to DBS (Alegret et al. 2004; Gaspari et al. 2006; Antonini et al. 2011; Merola et al. 2011; Elia et al. 2012; Dafsari et al. 2019; Liu et al. 2019). Likewise, CSFLI shows efficacy comparable to DBS (Weaver et al. 2005, 2012; Deuschl et al. 2020) for managing motor fluctuations/dyskinesias (Soileau et al. 2022). Although several open-label studies compared pump therapies and DBS (Alegret et al. 2004; Gaspari et al. 2006; Antonini et al. 2011; Merola et al. 2011; Elia et al. 2012; Dafsari et al. 2019; Liu et al. 2019), there are no head-to-head comparisons between these therapies and these studies did not include CSFLI. Similar to DBS non-levodopa-responsive symptoms may not benefit from pump therapies including CSFLI (Fasano et al. 2010; Fujimiya et al. 2011).

Besides motor symptoms, non-motor symptoms (NMS) vary across treatments, requiring their careful consideration (Leta et al. 2021). For instance, STN-DBS improves sleep, mood, cognition, perception, urogenital function, and excessive sweating (Dafsari et al. 2019; Petry-Schmelzer et al. 2019). LCIG benefits sleep, mood, and gastrointestinal function (Honig et al. 2009; Ricciardi et al. 2016; Antonini et al. 2017; Standaert et al. 2021; Diaconu et al. 2023). CSAI enhances mood, perception, attention, memory, and reduced excessive sweating (Reuter et al. 1999; Bhidayasiri et al. 2016; Borgemeester et al. 2016; Dafsari et al. 2019; Fernández-Pajarín et al. 2021). CSAI also improves urogenital function (Christmas et al. 1988; Martinez-Martin et al. 2011; Todorova and Chaudhuri 2013). Night-time CSAI shows benefits in sleep disorders (Cock et al. 2022). So far, no previous study addressed the effect of CSFLI in ameliorating NMS, emphasizing the need for further studies as NMS have a large impact on QoL in PD.

Recommendations for all DATs are derived from RCT data against best medical treatment, open-label studies comparing interventions, and expert consensus (Odin et al. 2015; Timpka et al. 2016; Antonini et al. 2018; Leta et al. 2021). While DBS is the most extensively studied intervention, infusion therapies incl. CSFLI provide effective alternatives. However, long-term efficacy and safety data are needed, ideally through head-to-head comparisons across different DAT options and in the context of combining different DATs (Pürner et al. 2023).

## Data Availability

Not applicable.
